# Overexpression of *GmGAMYB* Accelerates the Transition to Flowering and Increases Plant Height in Soybean

**DOI:** 10.3389/fpls.2021.667242

**Published:** 2021-05-10

**Authors:** Xue Yang, Xin Li, Jinming Shan, Yinghua Li, Yuntong Zhang, Yuhe Wang, Wenbin Li, Lin Zhao

**Affiliations:** Key Laboratory of Soybean Biology of Ministry of Education, Northeast Agricultural University, Harbin, China

**Keywords:** soybean, flowering time, plant height, *GmGAMYB*, gibberellin

## Abstract

The flowering time and plant height of soybean are important agronomic characters, which control the adaptability and yield of soybean. R2R3 MYB transcription factor plays an important regulatory role in plant growth and development. In this study, soybean *GmGAMYB* gene of R2R3-MYB type was induced by long-days (LDs). *GmGAMYB* showed higher transcriptional levels in the flowers, leaves and pods of soybean. Overexpression of *GmGAMYB* in transgenic soybean showed earlier flowering time and maturity in LDs and short-days (SDs). *GmGAMYB* interacted with *GmGBP1* and might promote flowering time by up-regulating the expression of *GmFULc* gene in soybean. Moreover, the expression level of *GmGAMYB* was also induced by gibberellins (GAs) and the plant height of *GmGAMYB-ox* plants was significantly increased, which was caused by the enlargement of internode cell in stem. Furthermore, *GmGAMYB* overexpression led to increased GA sensitivity in the hypocotyl of soybean seedlings compared with WT. *GmGAMYB* may be a positive regulator of GA response of promoting plant height by up-regulating the expression of *GmGA20ox* gene in soybean. Together, our studies preliminarily showed that the partial functions of *GmGAMYB* in regulating flowering time and GA pathway.

## Introduction

Soybean flowering time, maturity and plant height are the key factors affecting soybean adaptability and yield. Soybean [*Glycine max* (L.) Merrill] is a short-day (SD) plant, and its growth and development are very sensitive to photoperiod response. SD can promote flowering, and long-day (LD) inhibit the growth of flower bud (Kantolic and Slafer, 2007). This characteristic seriously hinders the adaptability of soybean varieties, and some soybean varieties planted in areas beyond their normal latitude of 2°N may significantly reduce their yields (Gai and Wang, 2001), so different types of varieties in photoperiod response are needed to adapt to different ecological conditions. Previous studies identified several major genetic loci affecting flowering and maturity in soybean, which have been designated as *E1* to *E11* and *J*, and several QTLs, such as *Tof11*/*Gp11*, *Tof12*/*Gp1*/*qFT12-1* (Bernard, 1971; Buzzell, 1971; Buzzell and Voldeng, 1980; Mcblain and Bernard, 1987; Ray et al., 1995; Bonato and Vello, 1999; Cober and Voldeng, 2001; Cober et al., 2010; Kong et al., 2014; Samanfar et al., 2017; Wang et al., 2019; Lu et al., 2020). Loss of function of the *E1*, *E3*, or *E4* alleles leads to photoperiod insensitivity and promotes early flowering under LDs (Liu et al., 2008; Watanabe et al., 2009; Xia et al., 2012). *E6* and *J* are primarily involved in promoting flowering under SDs (Ray et al., 1995; Bonato and Vello, 1999). Overexpression of *GmFT2a* and *GmFT5a*, two *FLOWERING LOCUS T* (*FT*) homologs, activated the expression of floral identity gene homologs such as *GmAP1*, *GmLFY* and *GmSOC1* to promote early flowering in soybean (Nan et al., 2014). In addition, two homologs of *SOC1*, *GmSOC1-like* and *GmSOC1*, had been isolated in soybean. Under LDs, *GmSOC1-like* overexpression promoted flowering in *Lotus corniculatus* (Na et al., 2013), while overexpression of *GmSOC1* saved the late flowering phenotype of *Arabidopsis soc1-1* mutants (Zhong et al., 2012). *GmAP1*, a *AP1* homologous gene in soybean, promotes early flowering and the alteration of floral organ patterns in tobacco (Chi et al., 2011).

Plant height of soybean is also an important agronomic character, which control the yield of soybean. Gibberellins (GAs) is one of the most important plant hormones in determining plant height (Helliwell et al., 1998; Ji et al., 2014). Recent studies have shown that *GmDW1* (dwarf mutant) encodes an ent-kaurene synthase (KS) and plays a key role in GA-regulated cell elongation in soybean stem internodes (Li et al., 2018). A homologous gene of *CCA1* and *LHY* in soybean, *GmLHY* encodes an MYB transcription factor, which affects plant height through mediating the GA pathway in soybean (Cheng et al., 2019). Despite the economic importance of soybean, the molecular mechanisms that regulate flowering and plant height are still poorly understood. Therefore, to explore new genes regulating soybean flowering and plant height, to further clarify the molecular mechanism of these genes involved in regulating flowering time, maturity and plant height, and to reduce the breeding pressure is a hot spot in the field of breeding.

R2R3-MYB transcription factors are associated with the regulation of plant morphology and metabolism, including embryonic cell development, tapetum and anther development (Higginson et al., 2003; Yang et al., 2007; Zhang et al., 2007), stomatal movement (Cominelli et al., 2005), glucoside biosynthesis (Gigolashvili et al., 2008), flavonoid accumulation (Stracke et al., 2007), trichome formation (Payne et al., 2000) and regulating flowering time (Seo et al., 2011; Liu et al., 2013), etc. R2R3-MYB transcription factors are classified into 22 subgroups according to the sequence conservatism of C-terminal region and GAMYB belongs to the 18th subgroup. GAMYB plays an important role in flowering induction, flower organ development, cereal seed germination and GA signaling pathway. In *Arabidopsis*, GAMYB-like genes *AtMYB33*, *AtMYB65*, and *AtMYB101* mediated GA signal transduction regulates petiole elongation and flowering response (Gocal et al., 2001). *AtMYB33* and *AtMYB65* is regulated by miR159 to promote programmed cell death and inhibit growth in aleurone (Alonso-Peral et al., 2010). In barley, *HvGAMYB* is upregulated by GA leading to a decrease in anther length and color (Murray et al., 2003). In rice, *OsGAMYB* functionally deficient mutants lead to abnormal development of stamens and anthers (Liu et al., 2010). Until now, the function of GAMYB members in soybean has been less reported.

Soybean *GAMYB* binding protein gene (*GmGBP1*), a SKIP homologous gene, functioned as a positive regulator of photoperiod control of flowering time and maturity responses (Zhao et al., 2018). Recent studies had preliminarily identified the interaction between *GmGBP1* and an R2R3-MYB soybean *GmGAMYB* gene through yeast two-hybrid system (Zhang et al., 2013). In the current study, *GmGAMYB* gene was cloned and its expression pattern under change of day length and GA treatments and biological function were characterized. Overexpression of *GmGAMYB* promoted soybean flowering time and maturity and increased plant height. The interaction between *GmGAMYB* and *GmGBP1* was verified by bimolecular fuorescent complimentary (BIFC) and Co-lmmunoprecipitation (Co-IP). Combined with RNA-Seq analysis, the overexpression of both genes regulated the expression of *GmFULc* gene. Therefore, we speculated that *GmGAMYB* and *GmGBP1* interacted to promote flowering time by upregulation of *GmFULc* gene expression in soybean. Moreover, RNA-seq analysis on *GmGAMYB-ox* soybean plants showed that GA synthetic gene *GmGA20ox* was up-regulated by *GmGAMYB*. *GmGAMYB* may be a positive regulator of GA response of promoting plant height by up-regulating the expression of *GmGA20ox* gene in soybean. These results preliminarily proposed the partial functions of *GmGAMYB* in regulating flowering time and GA pathway.

## Materials and Methods

### Plant Materials, Growth Conditions, and Records of Data

In this study, soybean “DongNong 50” was used as the wild-type (WT) control and the background plant for genetic transformation. “DongNong 42,” a photosensitive soybean variety was used to analyze the expression pattern of *GmGAMYB* gene. The seeds of two soybean cultivars were provided by the Northeast Agricultural University, Harbin, China.

For expression pattern analysis of *GmGAMYB* experiments, soybean “DongNong 42” were cultured at 25°C, 250 μmol m^–2^sec^–1^ white light, LD (16/8 h light/dark) conditions (LDs). A part of seedlings was transferred to SD (8/16 h light/dark) conditions (SDs) on day 15 after emergence. When the second trifoliate leaves were expanded, samples were taken every 3 h under LDs and SDs for a total of 24 h. Samples of different tissues including roots, stems, leaves, flowers, pods and seeds of soybean plants grown under LDs and SDs were collected. To analyze the response of *GmGAMYB* to GA_3_, 15-day-old seedlings under LDs as described above were sprayed with 100 μM GA_3_, and trifoliate leaves were sampled at 0, 0.5, 1, 3, 6, 9, 12, and 24 h after treatment. All samples were frozen in liquid nitrogen and stored at −80°C. Total RNA was extracted from all samples and the expression of *GmGAMYB* was analyzed by quantitative real-time RT-PCR (qRT-PCR).

In order to analyze *GmGAMYB* promoter activity in different *Arabidopsis* tissues and the activity of *GmGAMYB* promoter in *Arabidopsis* treated with GA_3_, Col-0 was used as the background plant for genetic transformation. Seeds of *proGmGAMYB:GUS* transgenic *Arabidopsis* were surface sterilized with 10% hypochlorite and then planted on MS agar medium. When *Arabidopsis* seedlings had two true leaves, they were transplanted into 1:1 of vermiculite and turfy-soil and cultured under LDs. When *Arabidopsis* seedlings had four leaves, some of them were soaked in 100 μM GA_3_ and sampled at 0, 3, and 6 h, respectively, for staining. On the 30^th^ day of culture, stem leaves, inflorescence, rosette leaves and roots of *Arabidopsis* plants were stained with X-Gluc staining solution. After 12 h at 37°C, then decolorized with 70% ethanol. After the chloroplast were removed, microscopic observation was carried out.

For statistical experiment of transgenic soybean maturity, T_3_ generation *GmGAMYB-ox-1*, *GmGAMYB-ox-2*, and WT soybean seeds were planted in plastic pots with dimensions of 30 cm high × 25 cm diameter at the top and 15 cm diameter at the bottom and cultured in a greenhouse at 25°C with 250 μmol m^–2^sec^–1^white light under LDs. The positive seedlings detected by Western blot were retained when the cotyledons fully developed. When the first trifoliate leaves were expanded, part of the seedlings were transferred to SDs under the same temperature regime. At least 15 plants of WT and two *GmGAMYB-ox* soybean lines were cultured under LDs and SDs, respectively. Five reproductive stages of soybean (R1, R2, R3, R5, and R7) were recorded according to the identification method of soybean growth period proposed by Fehr (Fehr et al., 1971). Period in which there was one flower at any node was R1. Period in which flowering at any of the two nodes with fully grown leaves in the uppermost part of the main stem was recorded as R2. Period in which pod was 0.5 cm (1/4 inch) long at any of the four uppermost nodes on the main stem with completely unrolled leaf was recorded as R3. Period in which seed 0.3 cm (1/8 inch) long in a pod at any of the four uppermost nodes on the main stem with completely unrolled leaf appeared was recorded as R5. Period in which a pod on the main stem reached its normal color at maturity was recorded as R7. At least 50% of the plants of each cultivar meet the criteria to be considered as reaching the specific R stage. At least 15 plants were analyzed each cultivar each time, and the experiments were repeated three times. Means ± SD deviation was used in the statistical analysis of the data.

### Plasmid Construction and Generation of Transgenic Plants

Firstly, the FLAG and HIS tag carrier were constructed by synthesizing the tandem repeats of 3 × FLAG and 6 × Histidine (3F6H) tags with *Not*I at the 5′end and *Xba*I at the 3′ end [5′-GCGGCCGCCCTGGAGCTCGGTACCCGGG(*Sma*I)GATCCCA GGATCT**GATTACAAGGATCATGATGGTGATTACAAGGAT CACGACATCGACTACAAGGATGACGATGACAAGCACCA TCATCACCACCATTGA**TCTCTAGA-3′, the sequences encoding 3F6H tag were in bold] (Song et al., 2012). The synthesized products containing 3F6H sequence at C terminus were cloned into *Not*I*-Xba*I sites of *pENTRY* vector (named *pENTRY-3F6H*). *GmGAMYB* gene fragment of 1602 bp was cloned from “DongNong 42” genome using *GmGAMYB*-*3F6H*-F and *GmGAMYB*-*3F6H*-R primers (Supplementary Table 2). The *GmGAMYB* gene fragment was reassembled by In-Fusion cloning system (Clontech, United States) connection onto the *pENTRY-3F6H* vector (named *35S:GmGAMYB-3F6H-pENTRY*). Recombinant plasmid *35S:GmGAMYB-3F6H-pENTRY* was synthesized into *pB7WG2* carrier by LR reaction (named *35S:GmGAMYB-3F6H-pB7WG2*). The construct was then transferred into *Agrobacterium tumefaciens* (*EHA105*). According to the method described previously (Zhao et al., 2018), transgenic soybean “DongNong 50” expressing *35S:GmGAMYB-3F6H-pB7WG2* was obtained. Transgenic soybean plants were screened by daubing 160 mg/L glufosinate into the preliminary leaves of the seedlings and further validated by PCR assay. Two most representative homozygous lines (*GmGAMYB-ox-1* and *GmGAMYB-ox-2*) were selected from five T_3_ transgenic soybean lines for further study.

The *GmGAMYB* genome sequence of 1945 bp in front of the 5′ untranslated region (UTR) served as the promoter region of the gene. The *GmGAMYB* promoter sequence was amplified from the genomic DNA of “DongNong 42” using *proGmGAMYB:GUS*-F and *proGmGAMYB:GUS*-R primers (Supplementary Table 2) and cloned into *pENTR/D-TOPO* (Life technologies) (named *proGmGAMYB-TOPO*). The recombinant plasmid was transferred to *pGWB533* vector through LR reaction (named *proGmGAMYB:GUS*), and then the new fusion vector was introduced into *Agrobacterium GV3101* for transforming into *Arabidopsis thaliana* (Col-0) using the floral dip method (Clough and Bent, 1998). Transformants were selected on MS agar medium with 5 mg/L hygromycin. T_3_ transgenic homozygous line seeds were selected for further study.

### Immunoblot Analysis

Using extraction buffer [150 mM NaCl, 50 mM Tris (pH 7.5), 10% glycerol, 5 mM EDTA, 0.5% Triton X-100, 0.5% (SDS), 1 mM DTT, 2 mM Na_3_VO_4_, 2 mM NaF and EDTA-free protease inhibitor tablet (Pierce)] to extract soybean protein to detect the protein expression of GmGAMYB driven by cauliflower-mosaic virus (CaMV) 35S promoter in transgenic soybean. Each 20 μg protein sample was subjected to 10% SDS-polyacrylamide gel electrophoresis, which was separated and transferred to nitrocellulose membrane. HRP-conjugated anti-FLAG antibody (A8592, Sigma) was used to detect 35S:GmGAMYB-3F6H protein. Mouse beta-actin monoclonal antibody (HRP-60008, Proteintech) was used to detect actin proteins as control. Super Signal West Pico Chemiluminescent substrate kits (Thermo Fisher Scientific) and the signal was detected by chemiluminescence imaging (Amersham Imager 600).

### Subcellular Localization of GmGAMYB

The *GmGAMYB* ORF sequence was amplified using *GmGAMYB-TOPO*-F and *GmGAMYB-TOPO*-R primers (Supplementary Table 2) and cloned into *pENTR/D-TOPO* (Life technologies) (named *GmGAMYB-TOPO*). The recombinant plasmid was transferred to *pGWB506* vector through LR reaction (named *35S: GmGAMYB-GFP*). The new fusion vector was introduced into *Agrobacterium* GV3101 for transforming into *N. benthamiana* (Hu et al., 2013). Red nuclear marker plasmid (H2B-RFP) was used to confirm the location of the cell nucleus (Goodin et al., 2002). The fluorescence signal was detected by fluorescence microscopy after 48 h tobacco leaves were infected.

### Cell Morphology Under Scanning Electron Microscopy

The internode cells of *GmGAMYB-ox* and WT soybeans were observed using an S-3400N scanning electron microscope (Hitachi Ltd., Tokyo, Japan) equipped with a cooling table.

### Hypocotyl Growth Assay of Seedlings

The seeds of *GmGAMYB-ox-1*, *GmGAMYB-ox-2* and WT were used to test the GA-mediated sensitivity of hypocotyl elongation. After normal germination on MS medium, all soybean seeds were transferred to MS medium containing 0 and 10 μM GA_3_, respectively.

### Endogenous GA_3_ Determination

*GmGAMYB-ox-1*, *GmGAMYB-ox-2*, and WT soybean seeds were cultured in a greenhouse at 25°C with 250 μmol m^–2^sec^–1^ white light under LDs. Leaf tissue (1 g fresh weight) was harvested from 20-day-old WT and *GmGAMYB-ox* seedlings. Plant GA_3_ ELISA Kit (Andy gene) was used to determine the endogenous GA_3_ levels in *GmGAMYB-ox* transgenic and WT soybean plants. The absorbance (OD) of the samples was measured at 450 nm with a microplate analyzer. The concentration of GA_3_ in the samples was calculated by the standard curve. At least six plants were analyzed each cultivar each time, and the experiments were repeated three times. Means ± SD deviation was used in the statistical analysis of the data.

### RNA-seq, Statistical Analysis and qRT-PCR Validation of Differentially Expressed Genes

The T_3_ generation *GmGAMYB-ox-1* and WT soybean seeds were cultured in the soil under LDs condition at 25°C. Trifoliate leaves of independent three 15-day-old WT and *GmGAMYB-ox-1* transgenic soybean seedlings were collected for each biological replicate, respectively, and the three biological replicates were used for RNA-seq analysis. The specific analysis method were described previously (Zhao et al., 2018). The cDNA library preparation, RNA-seq sequencing and assembly were performed on the Illumina sequencing platform (HiSeqTM 2000) by Beijing Genomics Institute, Shenzhen, China. Clean reads obtained after filtering the raw reads by removing adapter sequences and low-quality sequences are used for *de novo* assembly and read mapping of transcriptome. All Illumina reads produced by WT and *GmGAMYB-ox-1* by RNA-seq were compared in the reference genome annotation database of soybean^1^. Ratios of log_2_ were calculated with the reads per kilobase of exon model per million mapped reads (RPKM) value of every gene with *P*-value ≤ 0.001 and false discovery rate (FDR) ≤0.05 to determine the differentially expressed genes. For verification of differentially expressed genes, trifoliate leaves were harvested for qRT-PCR of WT and *GmGAMYB-ox* transgenic soybean plants the same as RNA-seq seedlings. Four differentially expressed genes associated with flowering time and three plant height related gene expression levels in *GmGAMYB-ox* transgenic soybean were further detected by qRT-PCR validation. Three biological replicates and three technical replicates were applied for the whole assays. Data shown are mean ± SD of three independent experiments (^∗∗^*P* < 0.01, Student’s *t*-test). Primers are listed in Supplementary Table 2.

### BIFC Assay

The constructed *GmGAMYB-TOPO* was transferred to the expression vector *pSITE-nEYFP-C1* through LR reaction (named as *35S:GmGAMYB-nYFP*). *GmGBP1-TOPO-F*, and *GmGBP1-TOPO-R* primers (Supplementary Table 2) were used for PCR amplification of *GmGBP1* gene cDNA fragment, which was cloned into *pENTR/D-TOPO* vector (named as *GmGBP1-TOPO*) and transferred to the expression vector *pSITE-cEYFP-C1* vector by LR reaction (named *35S:GmGBP1-cYFP*). All the above plasmids were introduced into *Agrobacterium* GV3101 for transforming into *N. benthamiana* (Hu et al., 2013). Red nuclear marker plasmid (H2B-RFP) was used to confirm the location of the cell nucleus. After infiltration, tobacco leaves were grown for 2 days, and YFP signals were detected by fluorescence microscope.

### Co-immunoprecipitation Assay

The constructed *GmGBP1-TOPO* was transferred to the expression vector *pGWB506* by LR reaction (named *35S:GmGBP1-GFP*). The constructed recombinant was introduced into *Agrobacterium* GV3101. Agrobacterium *35S:GmGAMYB-3F6H-pB7WG2* and *35S:GmGBP1-GFP* were individually or collectively transformed into *N. Benthamiana* leaves (Hu et al., 2013) and were sampled 2 days later. After protein extraction with a Co-IP buffer [50 mM Na-phosphate pH7.4, 135 mM NaCl, 4.7 mM KCl, 1 mM DTT, 50 μM MG-132, 2 mM Na_3_VO_4_, 2 mM NaF, and Complete protease inhibitor cocktail tablets (Roche)], 10 μl of Protein G-coupled magnetic beads (Dynabeads Protein G, Invitrogen) was used to capture anti-FLAG antibody (Sigma). After incubation at 4°C for 30 min, magnetic beads were washed three times for 5 min each time with 1 ml of Co-IP buffer without MG-132, Na_3_VO_4_, sodium fluoride, and protease inhibitor, and eluded with 2 × SDS sample buffer at 80°C for 5 min. At each sample, the immunoprecipitated proteins and 20 μl of the total extract were separated by 10% SDS-polyacrylamide gel electrophoresis gels and transferred to nitrocellulose membranes. Then, HRP-conjugated anti-FLAG antibody (A8592, Sigma) and HRP-conjugated anti-GFP Antibody (AB6663, Abcam) were applied to test GmGAMYB-FLAG and GmGBP1-GFP protein, respectively.

### Quantitative Real-Time RT-PCR Analysis

RNA isolation has been described previously (Zhao et al., 2013). qRT-PCR amplifications were performed using the TransStart^®^ Tip Green qPCR SuperMix (TransGen Biotech, Beijing) according to the manufacturer’s instructions on Applied Biosystems^TM^ 7500 Fast Dx Real-Time PCR Instrument (ABI). The PCR cycling conditions were as follows: 94°C for 30 sec; 40 cycles of 95°C for 5 sec and 60°C for 34 sec. *GmActin4* (GenBank accession number AF049106) was used as endogenous regulatory genes of soybean. The primers used in qRT-PCR analyses were shown in Supplementary Table 2. All experiments were performed at least three times with independent biological replicates.

## Results

### Sequence Analysis of the *GmGAMYB*

**FIGURE 1:**
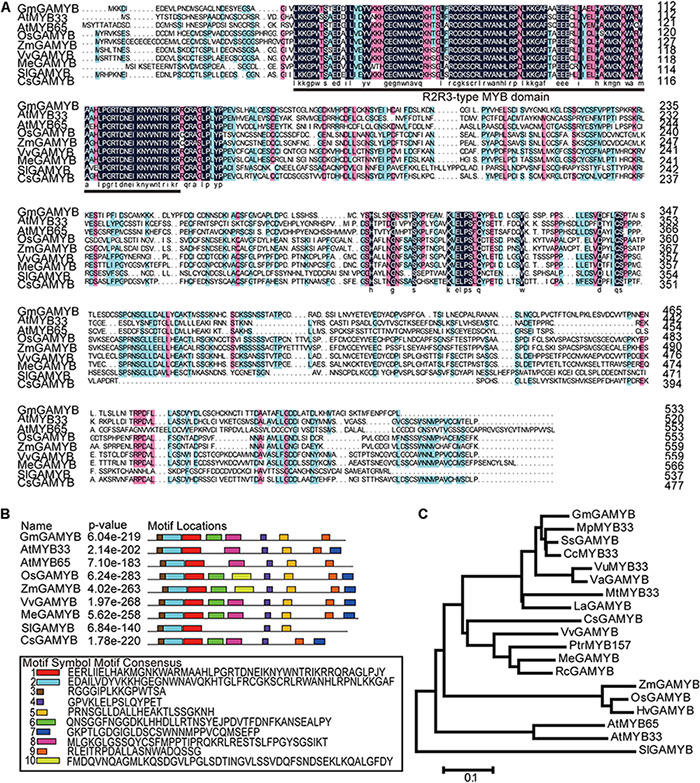
Sequence analysis of the *GmGAMYB*. **(A)** Multiple sequence alignment. Multiple sequence alignment of soybean GmGAMYB and GAMYB-like proteins of *Arabidopsis thaliana* (At), *Oryza sativa* (Os), *Zea mays* (Zm), *Vitis vinifera* (Vv), *Manihot esculenta* (Me), *Solanum lycopersicum* (Sl), and *Cucumis sativus* (Cs) (accession numbers are listed in Supplementary Table 1). The conserved R2R3-type domain is underlined. **(B)** Conservative Motif distribution of all GmGAMYB and GAMYB-Kike proteins using the MEME Suite, and ten motifs were identified. The sequences of the ten motifs are exhibited at the bottom. **(C)** Phylogenetic tree analysis was performed on GmGAMYB and proteins from other species with high similarity in NCBI. In addition to nine GAMYB-like proteins in the eight species mentioned above, *Mucuna pruriens* (Mp), *Spatholobus suberectus* (Ss), *Cajanus cajan* (Cc), *Vigna unguiculata* (Vu), *Vigna angularis* (Va), *Medicago truncatula* (Mt), *Lupinus angustifolius* (La), *Populus trichocarpa* (Ptr), *Ricinus communis* (Rc), *Hordeum vulgare* (Hv) were added to the construction of the phylogenetic tree. All the amino acid sequence information comes from the Phytozome database (accession numbers are listed in Supplementary Table 1). Phylogenetic tree was constructed using the neighbor joining method of MEGA 6.0.

The full-length cDNA sequence of *GmGAMYB* in the National Center for Biotechnology Information (NCBI) GenBank (Accession No. KC525897) was cloned from the leaves of “DongNong 42” by RT-PCR. The cDNA sequence of *GmGAMYB* is 2,975bp and contains 893bp 5′ UTR, 480bp 3′ UTR and 1,602 bp open reading frame, which encodes 533 amino acids with predicted molecular mass of 58.873 kDa. Multiple sequence alignment of soybean GmGAMYB and GAMYB-like proteins of *Arabidopsis thaliana*, *Oryza sativa*, *Zea mays*, *Vitis vinifera*, *Manihot esculenta*, *Solanum lycopersicum*, and *Cucumis sativus* showed that GmGAMYB protein contains a highly conserved R2R3 domain in the N-terminal (Figure 1A), which is the typical feature of R2R3-MYB subfamily. The conservative Motif distribution of all GmGAMYB and GAMYB-Like proteins showed Motif 1, Motif 2, and Motif 3 in the N-terminal, and a Motif 4 in the C-terminal of all proteins (Figure 1B), showing the highly conserved structural characteristics of GAMYB. Among them, the *Arabidopsis* GAMYB-like genes *AtMYB33* and *AtMYB65* have been reported to mediate GA signal transduction to regulate petiole elongation and flowering responses (Gocal et al., 2001). Phylogenetic tree analysis showed that GmGAMYB was located on the same branch with leguminous plants such as *Mucuna pruriens* (RDX95167.1), *Spatholobus suberectus* (TKY68413.1), *Cajanus cajan* (XP_020219565.1), *Vigna unguiculata* (XP_027909434.1), *Vigna angularis* (XP_017431637.1) and *Lupinus angustifolius* (XP_019449331.1) indicating that GmGAMYB proteins were relatively conservative in the evolution of leguminous plants (Figure 1C).

### GmGAMYB Protein Was Located in Cell Nucleus

**FIGURE 2:**
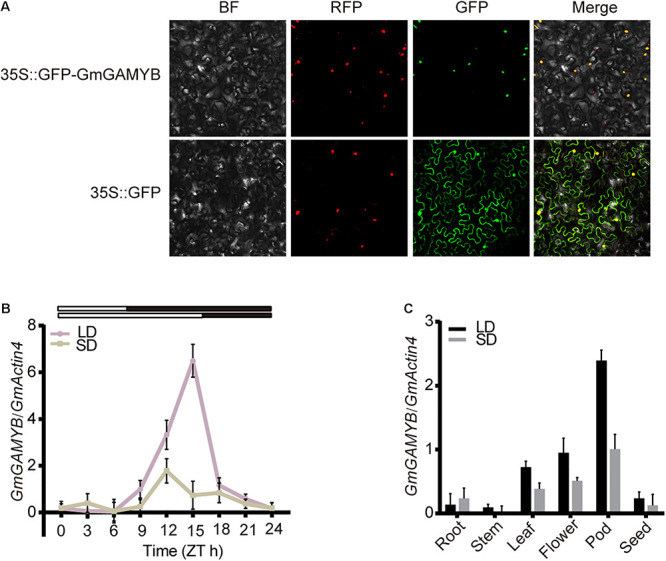
Subcellular localization and expression pattern analysis of *GmGAMYB*. **(A)** Subcellular localization of GmGAMYB protein. After infiltration, the tobacco leaves were grown for 2 days and the GFP signal was detected by fluorescence microscopy. Red nuclear marker plasmid (H2B-RFP) was used to confirm the location of the cell nucleus. GFP: Green Fluorescent Protein; RFP: Red Fluorescent Protein; BF: bright field; Merge: GFP, RFP and bright-field images. **(B)** The daily expression patterns of *GmGAMYB* in LDs and SDs. Soybean “Dongnong 42” plants, which were grown in LDs (16/8 h light/dark) for 15 days, were transferred to LDs or SDs (8/16 h light/dark) for 10 days for sampling at 3 h intervals. White and black bars at the top represented light and dark phases, respectively. **(C)** Tissue-specific expression of *GmGAMYB* under SDs and LDs. All the data were normalized with soybean *GmActin4* gene as internal reference. For each experiment, three technical replicates were conducted. Data shown are mean ± SD of three independent experiments.

The subcellular localization of the GmGAMYB protein might be crucial for its function. Fusion expression vector of *GmGAMYB* and green fluorescent protein gene (GFP) was constructed, and Agrobacterium-mediated transient expression of the fusion protein was transformed into tobacco leaves. Fluorescence microscope was used to observe green fluorescence in tobacco mesophyll cells. The observation results showed that the expression of 35S:GFP vector caused GFP fluorescence dispersed throughout the entire cell. In contrast, 35S: GFP-GmGAMYB fusion protein was specifically localized on the nucleus of tobacco mesophyll cells (Figure 2A). Red nuclear marker plasmid (H2B-RFP) was used to confirm the location of the cell nucleus. The results clearly showed that GmGAMYB was a nuclear-localization protein.

### Daylength Effect on Temporal and Spatial Expression Patterns of *GmGAMYB* in Soybean

The mRNA transcript abundance of *GmGAMYB* gene in “DongNong 42” leaves was analyzed within 24 h under SDs and LDs by qRT-PCR. The transcription abundance of *GmGAMYB* was significantly higher in LDs than in SDs, and reached the peak at 15 h after dawn, while it did not change much in SDs (Figure 2B). The results showed that *GmGAMYB* expression was induced by LDs in soybean leaves. Moreover, the expression levels of *GmGAMYB* gene in root, stem, leaf, flower, pod and seed of soybean tissues were also detected by LDs and SDs by qRT-PCR, respectively. The expression of *GmGAMYB* was highly expressed in leaves, flowers and pods, and higher in LDs than in SDs (Figure 2C). In addition, GUS histochemical staining was further used to detect the activity of *GmGAMYB* promoter in *Arabidopsis*. The stem leaves, inflorescence, rosette leaves and roots of transgenic *Arabidopsis* showed different degree of signal response after 30 days of growth, indicating that *GmGAMYB* promoter could be activated in different tissues of *Arabidopsis*, and its activation ability was stronger in inflorescence and stem leaves (Supplementary Figure 1). These results also suggested that *GmGAMYB* was induced by LD and possible involved in soybean growth and development.

### Overexpression of *GmGAMYB* Promoted Soybean Flowering Time and Maturity

**FIGURE 3:**
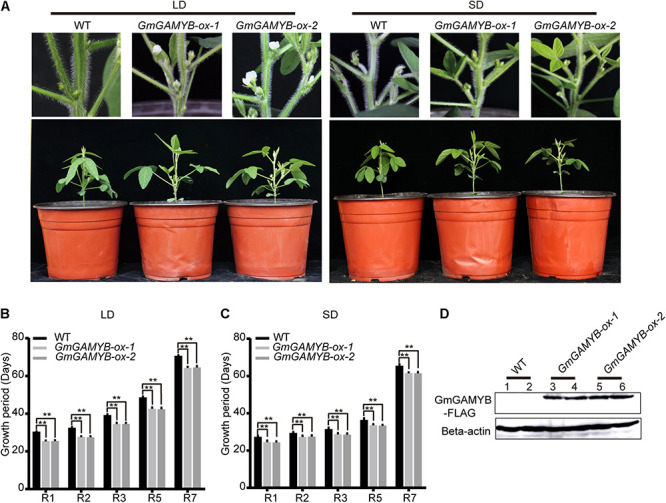
**(A)** Phenotypes of the T_3_ generation *GmGAMYB-ox* soybean in LDs and SDs at R1 stage. Phenotypes at R1 stage of wild-type (WT) and two *GmGAMYB-ox* transgenic soybean lines (*GmGAMYB-ox-1* and *GmGAMYB-ox-2*) in LDs and SDs. **(B)** Growth period of WT, *GmGAMYB-ox-1*, and *GmGAMYB-ox-2* in LDs. **(C)** Growth period of WT, *GmGAMYB-ox-1*, and *GmGAMYB-ox-2* in SDs. Data represent means ± SD of at least 15 seedlings. Asterisks indicate significant differences between *GmGAMYB-ox-1*, *GmGAMYB-ox-2* and WT (**P < 0.01, Student’s *t*-test). **(D)** Immunoblot analysis of *GmGAMYB-ox* transgenic soybean. 35S:GmGAMYB-3F6H protein expression in 15-day-old LD-grown transgenic seedlings. Beta-actin served as a loading control.

To further determine the biological function of *GmGAMYB* during the growth and development of soybean, the *35S:GmGAMYB-3F6H-pB7WG2* construct was transformed into soybean “DongNong 50.” Two representative *GmGAMYB-ox-1* and *GmGAMYB-ox-2* lines were selected from the transgenic lines for subsequent analysis of flowering time and maturation. Compared with WT, *GmGAMYB-ox* transgenic soybeans displayed earlier flowering and maturity under LDs and SDs (Figure 3A). The flowering time (R1) of *GmGAMYB-ox* plants was significantly earlier about 3 days under SDs and earlier about 5 days under LDs than WT plants. Furthermore, the R2, R3, R5, and R7 of the two *GmGAMYB-ox* soybean plants were also earlier than the WT plants, indicating that *GmGAMYB* shortened the whole maturity (Figures 3B,C). GmGAMYB (GmGAMYB-FLAG) protein with a size of 72KDa was detected by Western blot analysis in *GmGAMYB-ox-1* and *GmGAMYB-ox-2* transgenic soybeans (Figure 3D). In addition, *GmGAMYB-ox-1* and *GmGAMYB-ox-2* transgenic soybeans were identified at DNA and RNA levels (Supplementary Figures 2A,B). The results above indicated that the increase of *GmGAMYB* expression level promoted the flowering and maturity time.

### GmGAMYB Interacts With GmGBP1, the Ortholog of SKIP Protein in Soybean

**FIGURE 4:**
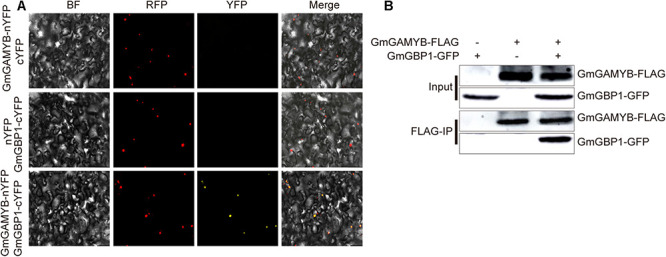
GmGAMYB interacts with GmGBP1. **(A)** BIFC assay for GmGAMYB interacts with GmGBP1. Leaves of *N. benthamiana* was co-transformed with GmGAMYB-nYFP and cYFP/GmGBP1-cYFP or nYFP and GmGBP1-cYFP. After infiltration, the tobacco leaves were grown for 2 days and the YFP signal was detected by fluorescence microscopy. Red nuclear marker plasmid (H2B-RFP) was used to confirm the location of the cell nucleus. YFP: Yellow Fluorescent Protein; RFP: Red Fluorescent Protein; BF: bright field; Merge: YFP, RFP and bright-field images. **(B)** Co-immunoprecipitation assays for GmGAMYB interact with GmGBP1. *35S:GmGAMYB-3F6H-pB7WG2* and *35S:GmGBP1-GFP* were individually or colle ctively transformed into *N. Benthamiana* leaves. GmGAMYB-FLAG protein was immunoprecipitated by anti-FLAG antibody, and then GmGAMYB-FLAG and GmGBP1-GFP protein in immunoprecipitated samples were detected by HRP coupled anti-FLAG antibody (Sigma) and HRP coupled anti-GFP antibody (Abcam), respectively.

Soybean *GmGBP1* gene is a ortholog of SKIP and had been functioned as a positive regulator of photoperiod mediated flowering pathway in tobaccos and *Arabidopsis* (Zhao et al., 2013) and photoperiod control of flowering time and maturity responses in soybean (Zhao et al., 2018). In addition, our previous studies had preliminarily confirmed the interaction between GmGAMYB and GmGBP1 through yeast two-hybrid system (Zhang et al., 2013). In this study, we further used BIFC and Co-IP to verify the occurrence of this interaction in plants. Strong fluorescence signals were observed in the nuclei of tobacco mesophyll cells co-transfected with *35S:GmGAMYB-nYFP* and *35S:GmGBP1-cYFP* (Figure 4A). However, it was not found in the cells transfected with vector control. GmGAMYB (GmGAMYB-FLAG) protein with 3 × FLAG was co-immunized to precipitate GmGBP1-GFP protein (Figure 4B). These results indicated that GmGAMYB interacted with GmGBP1 *in vitro* and *in vivo*. Therefore, we speculated that GmGAMYB might interact with GmGBP1 to regulate flowering time and maturation in the photoperiodic pathway.

### Identification of Differentially Expressed Genes Acting Downstream of *GmGAMYB* by RNA-Seq Analysis

The *GmGAMYB-ox* plants displayed earlier flowering time. In order to further understand the molecular network regulated by *GmGAMYB*, the global expression profiling of soybean genes in the leaves of 15-day-old *GmGAMYB-ox* and WT plants under LDs were compared by RNA-seq. Each individual sample generated about 44.54 million clean RNA-seq reads, of which 82.51% of the reads was mapped to the current soybean reference genome assembly. A total of 6643 differentially expressed genes (DEGs) between WT and the *GmGAMYB-ox* transgenic line were detected (Supplemental Data Set 1). These include 2,463 genes upregulated and 4,180 genes downregulated by *GmGAMYB* overexpression. | log_2_FC| >1 and *p* < 0.05 were used as criteria to screen out the genes with significant differences. Through functional analysis of plant differentially expressed genes of overexpressing *GmGAMYB* gene, it was found that four differentially expressed genes associated with flowering time and three plant height related gene were up-regulated by overexpression of *GmGAMYB* gene. *FRUITFULL* (*FUL*) genes are a group of downstream flowering genes that are known to play a major role in the reproductive transition. All three homologous genes *GmFULc* (*Glyma.05G018800*), *GmFUL1a (Glyma.04G159300)*, and *GmFUL2b* (*Glyma.17G081200*) in soybean are positively regulated by *GmGAMYB*. *GmFPF1* (*Glyma.04G074800*), a *FLOWERING PROMOTING FACTOR 1*, showed 35.32% amino acid identity with *AtFPF1* (*AT5G24860*), which promoted flowering in *Arabidopsis* (Kania et al., 1997), was also positively regulated by *GmGAMYB* in soybean.

**FIGURE 5:**
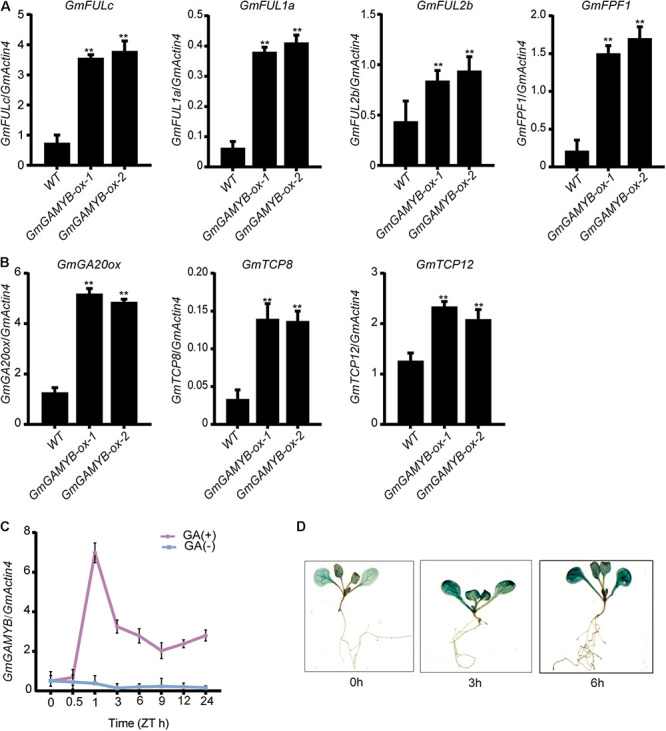
Validation of transcriptome sequencing results by qRT-PCR methods and the expression analysis of *GmGAMYB* treated with GA. **(A)** Relative expression of the *GmFULc*, *GmFUL1a*, *GmFUL2b*, and *GmFPF1* of *GmGAMYB-ox* and WT, respectively. **(B)** Relative expression of the *GmGA20ox*, *GmTCP8*, and *GmTCP12* of *GmGAMYB-ox* and WT, respectively. **(C)** The expression of *GmGAMYB* gene in soybean under gibberellin treatment. 15-day-old seedlings were sprayed with 100 μM GA_3_, and leaf samples were obtained at 0, 0.5, 1, 3, 6, 9, 12, and 24 h after treatment. **(D)** Gibberellin-treated transgenic *proGmGAMYB:GUS Arabidopsis* plant staining. When *Arabidopsis* grew to 4 leaves, some of them were soaked in 100 μM GA_3_ and sampled at 0, 3, and 6 h, respectively, for staining. For each experiment, three technical replicates were conducted. Data shown are mean ± SD of three independent experiments (**P < 0.01, Student’s *t*-test).

*GmGAMYB* positively regulated three genes related to plant height regulation: *Gibberellin 20-oxidase* (*GmGA20ox, Glyma.09G149200*), *GmTCP8* (*Glyma.05G027400*), and *GmTCP12* (*Glyma.06G193000*). *GA20ox* was a gene that regulated plant height in the GA metabolic pathway, and its overexpression saved the dwarfing phenotype in rice (Spielmeyer et al., 2002). *GmTCP8* and *GmTCP12*, members of the TEOSINTE-BRANCHED1/CYCLOIDEA/PCF (TCP) transcription factor family, are the best homologous matching of *AtTCP14* and *AtTCP15* in *Arabidopsis*, and their potential functions in regulating plant height (Davière et al., 2014; Feng et al., 2018). The relative expressions of *GmFULc*, *GmFUL1a*, *GmFUL2b*, *GmFPF1*, *GmGA20ox*, *GmTCP8*, and *GmTCP12* in *GmGAMYB-ox* leaves were higher than WT by qRT-PCR, which was consistent with the RNA-Seq data (Figures 5A,B).

### GmGAMYB Was Induced by Gibberellin

*GAMYB* has been demonstrated to respond to GA signal transduction in *Arabidopsis*, rice and Asian cotton (Achard et al., 2004; Fleet and Sun, 2005; Hartweck, 2008). The GA_3_-treated “DongNong 42” plants were sampled and the *GmGAMYB* level was analyzed by qRT-PCR to determine whether the biological function of *GmGAMYB* was related to the GA pathway. The results showed that the expression level of *GmGAMYB* in GA_3_-treated soybeans was most significantly up-regulated at 1 h, and the expression was also significantly up-regulated at other time points relative to the control (Figure 5C). In addition, GUS histochemical staining was also used to detect the activity of *GmGAMYB* promoter in *Arabidopsis* treated with GA_3_. The results showed that the response of *GmGAMYB* promoter after being treated with GA_3_ increased *GUS* gene expression (Figure 5D). These results indicated that *GmGAMYB* expression was positively regulated by GA_3_.

### Overexpression of *GmGAMYB* Increased Soybean Plant Height

**FIGURE 6:**
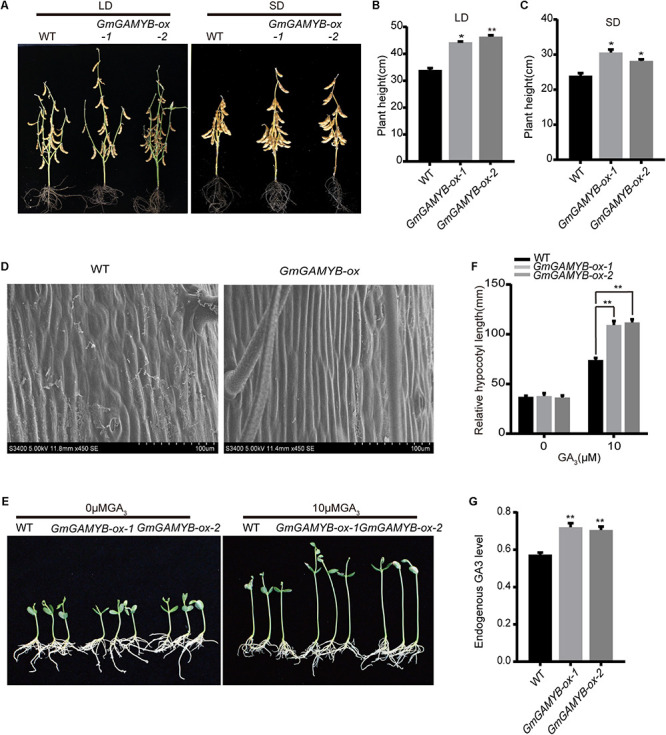
Phenotypes of the T_3_ generation *GmGAMYB-ox* soybean at R8 stage, internode epidermal cell morphology and the response of hypocotyl of seedlings to gibberellin. **(A)** Phenotypes at R8 stage of WT and two *GmGAMYB-ox* transgenic soybean lines (*GmGAMYB-ox-1* and *GmGAMYB-ox-2*) in LDs and SDs. **(B)** Plant height statistics of WT, *GmGAMYB-ox-1*, and *GmGAMYB-ox-2* in LDs. **(C)** Plant height statistics of WT, *GmGAMYB-ox-1* and *GmGAMYB-ox-2* in SDs. Data represent means ± SD of at least 15 seedlings. Asterisks indicate significant differences between *GmGAMYB-ox-1*, *GmGAMYB-ox-2* and WT (*P < 0.05 and **P < 0.01, Student’s *t*-test). **(D)** Cellular size analysis of WT and *GmGAMYB-ox* soybeans. Scanning electron microscope images of internode epidermal cells of WT and *GmGAMYB-ox* plants. Scale bars, 100 μm. **(E,F)** Hypocotyl lengths of 8-day-old soybeans in response to 0 and 10 μM GA_3_. Hypocotyl length was measured using ImageJ software. Check at least 20 seedlings per treatment. For each experiment, three technical replicates were conducted. **(G)** Determination of endogenous GA_3_ levels in the leaves of 20-day-old WT and *GmGAMYB-ox* plants. At least six plants were analyzed each cultivar each time and the experiments were repeated three times. Data shown are mean ± SD of three independent experiments (**P < 0.01, Student’s *t*-test).

The plant height of *GmGAMYB-ox* plants was significantly increased compared with WT under both LDs and SDs (Figures 6A–C). The stem epidermal cells of *GmGAMYB-ox* and WT plants were selected for scanning microscope examination to investigate the reason why the plant height of *GmGAMYB-ox* transgenic soybean was higher than WT. The results showed that the internode epidermal cells of *GmGAMYB-ox* soybean were significantly larger than those of WT in longitudinal direction. Therefore, the higher phenotype of *GmGAMYB-ox* soybean was due to the internode cell enlargement in stem (Figure 6D). Previous studies have shown that GA is one of the most important hormones in determining plant height (Jing et al., 2019). The results above also showed that *GmGAMYB* was positively regulated by GAs, so it was speculated that the change in *GmGAMYB-ox* transgenic soybean plant height was related to GA signal pathway.

### Response of Hypocotyl of *GmGAMYB-ox* Transgenic Seedlings to Gibberellin and Endogenous GA_3_ Determination

To test the response of *GmGAMYB* to GA, *GmGAMYB-ox* transgenic soybeans and WT were treated with GA_3_. The results showed that exogenous GA_3_ could increase the hypocotyl length of *GmGAMYB-ox* and WT seedlings, and the promotion effect of GA_3_ on the hypocotyl of *GmGAMYB-ox* seedlings was more obvious than WT (Figures 6E,F). Therefore, *GmGAMYB* overexpression led to increased GA sensitivity in the hypocotyl of soybean seedlings compared with WT. Detection of the endogenous GA_3_ levels of WT and *GmGAMYB-ox* soybean plants showed that the endogenous GA_3_ level in *GmGAMYB-ox* soybean plants was significantly higher than that in WT (Figure 6G). These findings indicated that the *GmGAMYB-ox* has a high active gibberellin level and *GmGAMYB* might positively regulate GA biosynthesis, thereby limiting soybean plant height.

## Discussion

In plants, the MYB gene encodes one of the largest transcription factor families. The MYB protein family has a typical conserved MYB domain, and the two duplicated MYB domains are named 2R-MYB (R2R3-MYB) (Dubos et al., 2010). R2R3 MYB transcription factor plays an important regulatory role in plant growth and development. There were 244 R2R3-MYB genes identified among 252 MYB transcription factors in soybean (Du et al., 2012) and 17 members have been reported so far (Yang et al., 2018). For example, the silencing of soybean *GmMYB-G20-1* can change the color of soybean flowers, which may be similar to *W2* gene (Takahashi et al., 2013). *GmMYBJ1* overexpression enhanced the tolerance of *Arabidopsis* to drought and low temperature stress (Su et al., 2014). *GmMYB73* promotes lipid accumulation, elevate seed size and thousand-seed weights in transgenic *Arabidopsis* (Liu et al., 2014). A specific seed coat expression R2R3 MYB gene (*Glyma09g36990*) was identified by fine mapping, affecting brown seed coat/hilum phenotype in soybean (Gillman et al., 2011). Overexpression of *GmMYB181* caused phenotypic changes in *Arabidopsis* including flower organ morphology, plant structure and fruit size (Yang et al., 2018). However, relatively few of R2R3MYB transcription factor members in soybean have been studied in regulating flowering time.

In this study, *GmGAMYB*, a new MYB transcription factor, was isolated from soybean and identified. GmGAMYB was identified as a member of the GAMYB subfamily of R2R3 MYB transcription factors by amino acid sequence alignment with typical GAMYB-like proteins in several species and conservative motif analysis. According to the subcellular localization of GmGAMYB in tobacco leaf cells, the GmGAMYB-GFP fusion protein was specifically localized on the nucleus of tobacco leaf cells, which indicated that GmGAMYB is a nuclear localization protein that matches its function as a transcription factor. Soybean is a typical SD plant, which is particularly sensitive to photoperiod. Photoperiod responses lay the foundation for the adaptation of different soybean varieties and play important roles in flowering and maturation of soybean (Hartwig, 1970). Recently, it has been reported that the R2R3 MYB transcription factor is also involved in the control of flowering time. *WEREWOLF* (*WER*), encodes an R2R3 MYB transcription factor, expressed in the epidermis of leaves and regulated flowering time through photoperiod pathway in *Arabidopsis* (Seo et al., 2011). In *Populus*, R2R3 MYB transcription factor gene *ptrMYB192* was highly expressed in Populus mature leaves and overexpression of *ptrMYB192* delayed flowering time in *Arabidopsis* (Liu et al., 2013). *GmGAMYB* mRNA was higher expressed in flowers, leaves and pods of soybean, suggesting that *GmGAMYB* may be involved in soybean growth and development. The analysis of daily expression pattern of *GmGAMYB* showed that the expression level of *GmGAMYB* was induced by LDs. Overexpression of *GmGAMYB* in transgenic soybean showed early flowering time and maturity in LDs and SDs, but this phenotype was more obvious in LDs than in SDs. Therefore, *GmGAMYB* may be involved in the photoperiod regulation of soybean flowering.

In addition, we previously reported that *GmGBP1* might be a positive regulator upstream of *GmFT2a* and *GmFT5a* to activate the expression of *GmFULc* to promote flowering (Zhao et al., 2018). Our previous studies had preliminarily confirmed the interaction between GmGAMYB and GmGBP1 through yeast two-hybrid system (Zhang et al., 2013). In this study, the interaction between GmGAMYB and GmGBP1 was further verified by BiFC and Co-IP assays. Therefore, we inferred that *GmGAMYB* might interact with *GmGBP1* to induce the expression of *GmFULc* to promote flowering time and maturity in soybean. *FRUITFULL (FUL)*, a family of MADS-box transcription factor protein genes, is a major of downstream flowering genes, which is known to play an important role in reproductive transition. In the photoperiod-dependent flowering pathway of *Arabidopsis thaliana*, the *Arabidopsis* flowering integrator *FT* promotes the transition to flowering by regulating the accumulation of *FUL* in *Arabidopsis* leaves (Teper-Bamnolker and Samach, 2005). Soybean *GmFT1a* inhibited the expression of *GmFULa* (a soybean FUL homolog) and delayed flowering (Liu et al., 2018). In the current study, RNA-seq analysis of *GmGAMYB* overexpression in soybean showed that three members of soybean *FUL* gene family (*GmFULc*, *GmFUL1a*, and *GmFUL2b*) were significantly upregulated. *GmGBP1* is induced by SDs and is a positive regulator of photoperiod control of flowering time (Zhao et al., 2018), while *GmGAMYB* is induced by LDs and its overexpression also promotes soybean flowering. Therefore, the final verification of how these two genes to regulate soybean flowering is the focus of follow-up research.

In crop breeding, plant height is generally regarded as a central yield trait (Reinhardt and Kuhlemeier, 2002). GAs is one of the most important plant hormones in determining plant height (Helliwell et al., 1998; Ji et al., 2014). GAs plays a physiological role in regulating plant growth and development. GAs is involved in seed germination (Debeaujon and Koornneef, 2000) and stem elongation (Luo et al., 2006), xylem synthesis (Mauriat and Moritz, 2009), hypocotyls elongation (Coles et al., 1999), etc. In this study, *GmGAMYB* was induced and the hypocotyls of *GmGAMYB-ox* transgenic soybean seedlings were significantly longer than those of WT seedlings by exogenous GA_3_ treatment. The results showed that the *GmGAMYB-ox* transgenic soybean was more sensitive to GA than WT, and *GmGAMYB* was a positive response factor of GA pathway. The plant height of *GmGAMYB-ox* was significantly higher than that of WT in LDs and SDs. *GmGA20ox* was up-regulated by *GmGAMYB* to increase plant height. In many species, the overexpression of *GA20ox* can change the phenotype of plants and is a key enzyme for the synthesis of bioactive GA. The expression of *ZmGA20ox* cDNA in switchgrass increased the bioactive GA level, making the internodes and leaves longer (Do et al., 2016). Overexpression of *StGA20ox1* encoding potato GA20ox resulted in increased plant height and petiole growth in potato (Carrera et al., 2000). Therefore, we speculated that *GmGAMYB* was a positive response factor of GA pathway, which increased the plant height of soybean by inducing the expression of *GmGA20ox*. These results preliminarily proposed the partial functions of *GmGAMYB* in regulating flowering time and GA pathway, providing a certain theoretical basis for the subsequent application of *GmGAMYB* in soybean breeding and agricultural production.

## Data Availability Statement

The datasets presented in this study can be found in online repositories. The names of the repository/repositories and accession number(s) can be found below: NCBI-SRA database under the BioProject no. PRJNA683993 and accession nos. SRR13241701, SRR13241702, SRR13241703, SRR13241704, SRR13241705, and SRR13241706 for the RNA-seq data.

## Author Contributions

XY performed protein interaction and data analysis. XL and JS performed phenotypic observation and measurement. YL performed gene cloning transformation. YZ and YW performed cell morphology detection and RNA data analysis. XY, LZ, and WL wrote the manuscript. All authors contributed to the article and approved the submitted version.

## Conflict of Interest

The authors declare that the research was conducted in the absence of any commercial or financial relationships that could be construed as a potential conflict of interest.

## Footnotes

1 http://www.phytozome.net

## References

[B1] Achard P. Herr A. Baulcombe D. Harberd N. (2004). Modulation of floral development by a gibberellin-regulated microRNA. *Development (Cambridge, England)* 131 3357–3365. 10.1242/dev.01206 15226253

[B2] Alonso-Peral M. Li J. Li Y. Allen R. Schnippenkoetter W. Ohms S. (2010). The microRNA159-regulated GAMYB-like genes inhibit growth and promote programmed cell death in *Arabidopsis*. *Plant Physiol.* 154 757–771. 10.1104/pp.110.160630 20699403 PMC2949021

[B3] Bernard R. L. (1971). Two major genes for time of flowering and maturity in soybeans1. *Crop Sci.* 11 242–244. 10.2135/cropsci1971.0011183x001100020022x

[B4] Bonato E. R. Vello N. A. (1999). E6, a dominant gene conditioning early flowering and maturity in soybeans. *Genet. Mol. Biol.* 22 229–232. 10.1590/S1415-47571999000200016

[B5] Buzzell R. I. (1971). Inheritance of a soybean flowering response to fluorescent-daylength conditions. *Can. J. Genet. Cytol.* 13 703–707. 10.1080/00222937100770511

[B6] Buzzell R. I. Voldeng H. D. (1980). Inheritance of insensitivity to long daylength. *Soybean Genet. Newsl.* 7 26–29.

[B7] Carrera E. Bou J. García-Martínez J. Prat S. (2000). Changes in GA 20-oxidase gene expression strongly affect stem length, tuber induction and tuber yield of potato plants. *Plant J.* 22 247–256. 10.1046/j.1365-313x.2000.00736.x 10849342

[B8] Cheng Q. Dong L. Su T. Li T. Gan Z. Nan H. (2019). CRISPR/Cas9-mediated targeted mutagenesis of GmLHY genes alters plant height and internode length in soybean. *BMC Plant Biol.* 19:562. 10.1186/s12870-019-2145-8 31852439 PMC6921449

[B9] Chi Y. Huang F. Liu H. Yang S. Yu D. (2011). An APETALA1-like gene of soybean regulates flowering time and specifies floral organs. *J. Plant Physiol.* 168 2251–2259. 10.1016/j.jplph.2011.08.007 21963279

[B10] Clough S. Bent A. (1998). Floral dip: a simplified method for Agrobacterium-mediated transformation of *Arabidopsis thaliana*. *Plant J.* 16 735–743. 10.1046/j.1365-313x.1998.00343.x 10069079

[B11] Cober E. R. Molnar S. J. Charette M. Vol De Ng H. D. (2010). A new locus for early maturity in soybean. *Crop Sci.* 50 524–527. 10.2135/cropsci2009.04.0174

[B12] Cober E. R. Voldeng H. D. (2001). A new soybean maturity and photoperiod-sensitivity locus linked to E1 and T. *Crop Sci.* 41 698–701. 10.2135/cropsci2001.413698x

[B13] Coles J. Phillips A. Croker S. García-Lepe R. Lewis M. Hedden P. (1999). Modification of gibberellin production and plant development in *Arabidopsis* by sense and antisense expression of gibberellin 20-oxidase genes. *Plant J.* 17 547–556. 10.1046/j.1365-313x.1999.00410.x 10205907

[B14] Cominelli E. Galbiati M. Vavasseur A. Conti L. Sala T. Vuylsteke M. (2005). A guard-cell-specific MYB transcription factor regulates stomatal movements and plant drought tolerance. *Curr. Biol. CB* 15 1196–1200. 10.1016/j.cub.2005.05.048 16005291

[B15] Davière J. Wild M. Regnault T. Baumberger N. Eisler H. Genschik P. (2014). Class I TCP-DELLA interactions in inflorescence shoot apex determine plant height. *Curr. Biol. CB* 24 1923–1928. 10.1016/j.cub.2014.07.012 25127215

[B16] Debeaujon I. Koornneef M. (2000). Gibberellin requirement for *Arabidopsis* seed germination is determined both by testa characteristics and embryonic abscisic acid. *Plant Physiol.* 122 415–424. 10.1104/pp.122.2.415 10677434 PMC58878

[B17] Do P. De Tar J. Lee H. Folta M. Zhang Z. (2016). Expression of ZmGA20ox cDNA alters plant morphology and increases biomass production of switchgrass (*Panicum virgatum* L.). *Plant Biotechnol. J.* 14 1532–1540. 10.1111/pbi.12514 26801525 PMC5066678

[B18] Du H. Yang S. Liang Z. Feng B. Liu L. Huang Y. (2012). Genome-wide analysis of the MYB transcription factor superfamily in soybean. *BMC Plant Biol.* 12:106. 10.1186/1471-2229-12-106 22776508 PMC3462118

[B19] Dubos C. Stracke R. Grotewold E. Weisshaar B. Martin C. Lepiniec L. (2010). MYB transcription factors in *Arabidopsis*. *Trends Plant Sci.* 15 573–581. 10.1016/j.tplants.2010.06.005 20674465

[B20] Fehr W. R. Caviness C. E. Burmood D. T. Pennington J. S. (1971). Stage of development descriptions for soybeans, *Glycine max* (L) Merrill. *Crop Sci.* 11 929–931. 10.2135/cropsci1971.0011183x001100060051x

[B21] Feng Z. Xu S. Liu N. Zhang G. Hu Q. Gong Y. (2018). Soybean TCP transcription factors: evolution, classification, protein interaction and stress and hormone responsiveness. *Plant Physiol. Biochem. PPB* 127 129–142. 10.1016/j.plaphy.2018.03.020 29579640

[B22] Fleet C. Sun T. (2005). A DELLAcate balance: the role of gibberellin in plant morphogenesis. *Curr. Opin. Plant Biol.* 8 77–85. 10.1016/j.pbi.2004.11.015 15653404

[B23] Gai J. Y. Wang Y. S. (2001). A study on the varietal eco-regions of soybeans in China. *Sci. Agric. Sin.* 34 139–145.

[B24] Gigolashvili T. Engqvist M. Yatusevich R. Müller C. Flügge U. (2008). HAG2/MYB76 and HAG3/MYB29 exert a specific and coordinated control on the regulation of aliphatic glucosinolate biosynthesis in *Arabidopsis thaliana*. *New Phytol.* 177 627–642. 10.1111/j.1469-8137.2007.02295.x 18042203

[B25] Gillman J. Tetlow A. Lee J. Shannon J. Bilyeu K. (2011). Loss-of-function mutations affecting a specific Glycine max R2R3 MYB transcription factor result in brown hilum and brown seed coats. *BMC Plant Biol.* 11:155. 10.1186/1471-2229-11-155 22070454 PMC3229458

[B26] Gocal G. Sheldon C. Gubler F. Moritz T. Bagnall D. MacMillan C. (2001). GAMYB-like genes, flowering, and gibberellin signaling in *Arabidopsis*. *Plant Physiol.* 127 1682–1693. 10.1104/pp.127.4.1682 11743113 PMC133573

[B27] Goodin M. Dietzgen R. Schichnes D. Ruzin S. Jackson A. (2002). pGD vectors: versatile tools for the expression of green and red fluorescent protein fusions in agroinfiltrated plant leaves. *Plant J.* 31 375–383. 10.1046/j.1365-313x.2002.01360.x 12164816

[B28] Hartweck L. (2008). Gibberellin signaling. *Planta* 229 1–13. 10.1007/s00425-008-0830-1 18936962

[B29] Hartwig E. E. (1970). Growth and reproductive characteristics of soybeans (Glycine max (L.) Merr.) grown under short-day conditions. *Trop. Sci.* 12 47–53.

[B30] Helliwell C. Sheldon C. Olive M. Walker A. Zeevaart J. Peacock W. (1998). Cloning of the *Arabidopsis* ent-kaurene oxidase gene GA3. *Proc. Natl. Acad. Sci. U.S.A.* 95 9019–9024. 10.1073/pnas.95.15.9019 9671797 PMC21195

[B31] Higginson T. Li S. Parish R. (2003). AtMYB103 regulates tapetum and trichome development in *Arabidopsis thaliana*. *Plant J.* 35 177–192. 10.1046/j.1365-313x.2003.01791.x 12848824

[B32] Hu Y. Chen L. Wang H. Zhang L. Wang F. Yu D. (2013). Arabidopsis transcription factor WRKY8 functions antagonistically with its interacting partner VQ9 to modulate salinity stress tolerance. *Plant J.* 74 730–745. 10.1111/tpj.12159 23451802

[B33] Ji S. Gururani M. Lee J. Ahn B. Chun S. (2014). Isolation and characterisation of a dwarf rice mutant exhibiting defective gibberellins biosynthesis. *Plant Biol. (Stuttgart, Germany)* 16 428–439. 10.1111/plb.12069 23944972

[B34] Jing Y. Zhao X. Wang J. Lian M. Li W. (2019). Identification of loci and candidate genes for plant height in soybean (Glycine max) via genome−wide association study. *Plant Breed.* 138 721–732. 10.1111/pbr.12735

[B35] Kania T. Russenberger D. Peng S. Apel K. Melzer S. (1997). FPF1 promotes flowering in *Arabidopsis*. *Plant Cell* 9 1327–1338. 10.1105/tpc.9.8.1327 9286110 PMC157001

[B36] Kantolic A. Slafer G. (2007). Development and seed number in indeterminate soybean as affected by timing and duration of exposure to long photoperiods after flowering. *Ann. Bot.* 99 925–933. 10.1093/aob/mcm033 17452381 PMC2802919

[B37] Kong F. J. Nan H. Y. Cao F. F. Li Y. Wu F. Wang J. (2014). A new dominant gene E9 conditions early flowering and maturity in soybean. *Crop Sci.* 54 2529–2535. 10.2135/cropsci2014.03.0228

[B38] Li Z. Guo Y. Ou L. Hong H. Wang J. Liu Z. (2018). Identification of the dwarf gene GmDW1 in soybean (Glycine max L.) by combining mapping-by-sequencing and linkage analysis. *TAG Theor. Appl. Genet.* 31 1001–1016. 10.1007/s00122-017-3044-8 29550969 PMC5895683

[B39] Liu B. Kanazawa A. Matsumura H. Takahashi R. Harada K. Abe J. (2008). Genetic redundancy in soybean photoresponses associated with duplication of the phytochrome A gene. *Genetics* 180 995–1007. 10.1534/genetics.108.092742 18780733 PMC2567397

[B40] Liu S. Wang X. Li E. Douglas C. J. Chen J. G. Wang S. (2013). R2R3 MYB transcription factor PtrMYB192 regulates flowering time in *Arabidopsis* by activating FLOWERING LOCUS C. *J. Plant Biol.* 56 243–250. 10.1007/s12374-013-0135-1

[B41] Liu W. Jiang B. Ma L. Zhang S. Zhai H. Xu X. (2018). Functional diversification of Flowering Locus T homologs in soybean: GmFT1a and GmFT2a/5a have opposite roles in controlling flowering and maturation. *New Phytol.* 217 1335–1345. 10.1111/nph.14884 29120038 PMC5900889

[B42] Liu Y. Li Q. Lu X. Song Q. Lam S. Zhang W. (2014). Soybean GmMYB73 promotes lipid accumulation in transgenic plants. *BMC Plant Biol.* 14:73. 10.1186/1471-2229-14-73 24655684 PMC3998039

[B43] Liu Z. Bao W. Liang W. Yin J. Zhang D. (2010). Identification of gamyb-4 and analysis of the regulatory role of GAMYB in rice anther development. *J. Integr. Plant Biol.* 52 670–678. 10.1111/j.1744-7909.2010.00959.x 20590996

[B44] Lu S. Dong L. Fang C. Liu S. Kong L. Cheng Q. (2020). Stepwise selection on homeologous PRR genes controlling flowering and maturity during soybean domestication. *Nat. Genet.* 52 428–436. 10.1038/s41588-020-0604-7 32231277

[B45] Luo A. Qian Q. Yin H. Liu X. Yin C. Lan Y. (2006). EUI1, encoding a putative cytochrome P450 monooxygenase, regulates internode elongation by modulating gibberellin responses in rice. *Plant Cell Physiol.* 47 181–191. 10.1093/pcp/pci233 16306061

[B46] Mauriat M. Moritz T. (2009). Analyses of GA20ox- and GID1-over-expressing aspen suggest that gibberellins play two distinct roles in wood formation. *Plant J.* 58 989–1003. 10.1111/j.1365-313X.2009.03836.x 19228336

[B47] Mcblain B. Bernard R. (1987). A new gene affecting the time of flowering and maturity in soybeans. *J. Heredity* 78 160–162. 10.1093/oxfordjournals.jhered.a110349

[B48] Murray F. Kalla R. Jacobsen J. Gubler F. (2003). A role for HvGAMYB in anther development. *Plant J.* 33 481–491. 10.1046/j.1365-313x.2003.01641.x 12581306

[B49] Na X. Jian B. Yao W. Wu C. Hou W. Jiang B. (2013). Cloning and functional analysis of the flowering gene GmSOC1-like, a putative SUPPRESSOR OF OVEREXPRESSION CO1/AGAMOUS-LIKE 20 (SOC1/AGL20) ortholog in soybean. *Plant Cell Reports* 32 1219–1229. 10.1007/s00299-013-1419-0 23636663

[B50] Nan H. Cao D. Zhang D. Li Y. Lu S. Tang L. (2014). GmFT2a and GmFT5a redundantly and differentially regulate flowering through interaction with and upregulation of the bZIP transcription factor GmFDL19 in soybean. *PLoS One* 9:e97669. 10.1371/journal.pone.0097669 24845624 PMC4028237

[B51] Payne C. Zhang F. Lloyd A. (2000). GL3 encodes a bHLH protein that regulates trichome development in arabidopsis through interaction with GL1 and TTG1. *Genetics* 156 1349–1362. 11063707 10.1093/genetics/156.3.1349 PMC1461316

[B52] Ray J. D. Hinson K. Mankono J. Malo M. F. (1995). Genetic control of a long-juvenile trait in soybean. *Crop Sci.* 35 1001–1006. 10.2135/cropsci1995.0011183X003500040012x

[B53] Reinhardt D. Kuhlemeier C. (2002). Plant architecture. *EMBO Rep.* 3 846–851. 10.1093/embo-reports/kvf177 12223466 PMC1084230

[B54] Samanfar B. Molnar S. Charette M. Schoenrock A. Dehne F. Golshani A. (2017). Mapping and identification of a potential candidate gene for a novel maturity locus, E10, in soybean. *TAG. Theoretical and applied genetics*. *Theor. Angewandte Genet.* 130 377–390. 10.1007/s00122-016-2819-7 27832313

[B55] Seo E. Yu J. Ryu K. Lee M. Lee I. (2011). WEREWOLF, a regulator of root hair pattern formation, controls flowering time through the regulation of FT mRNA stability. *Plant Physiol.* 156 1867–1877. 10.1104/pp.111.176685 21653190 PMC3149934

[B56] Song Y. Smith R. To B. Millar A. Imaizumi T. (2012). FKF1 conveys timing information for CONSTANS stabilization in photoperiodic flowering. *Science (New York, N.Y.)* 336 1045–1049. 10.1126/science.1219644 22628657 PMC3737243

[B57] Spielmeyer W. Ellis M. Chandler P. (2002). Semidwarf (sd-1), “green revolution” rice, contains a defective gibberellin 20-oxidase gene. *Proc. Natl. Acad. Sci. U.S.A.* 99 9043–9048. 10.1073/pnas.132266399 12077303 PMC124420

[B58] Stracke R. Ishihara H. Huep G. Barsch A. Mehrtens F. Niehaus K. (2007). Differential regulation of closely related R2R3-MYB transcription factors controls flavonol accumulation in different parts of the *Arabidopsis thaliana* seedling. *Plant J.* 50 660–677. 10.1111/j.1365-313X.2007.03078.x 17419845 PMC1976380

[B59] Su L. Li J. Liu D. Zhai Y. Zhang H. Li X. (2014). A novel MYB transcription factor, GmMYBJ1, from soybean confers drought and cold tolerance in *Arabidopsis thaliana*. *Gene* 538 46–55. 10.1016/j.gene.2014.01.024 24440241

[B60] Takahashi R. Yamagishi N. Yoshikawa N. (2013). A MYB transcription factor controls flower color in soybean. *J. Heredity* 104 149–153. 10.1093/jhered/ess081 23048163

[B61] Teper-Bamnolker P. Samach A. (2005). The flowering integrator FT regulates SEPALLATA3 and FRUITFULL accumulation in *Arabidopsis* leaves. *Plant Cell* 17 2661–2675. 10.1105/tpc.105.035766 16155177 PMC1242264

[B62] Wang F. Nan H. Chen L. Fang C. Lu S. (2019). A new dominant locus, E11, controls early flowering time and maturity in soybean. *Mol. Breed.* 39:70. 10.1007/s11032-019-0978-3

[B63] Watanabe S. Hideshima R. Xia Z. Tsubokura Y. Sato S. Nakamoto Y. (2009). Map-based cloning of the gene associated with the soybean maturity locus E3. *Genetics* 182 1251–1262. 10.1534/genetics.108.098772 19474204 PMC2728863

[B64] Xia Z. Watanabe S. Yamada T. Tsubokura Y. Nakashima H. Zhai H. (2012). Positional cloning and characterization reveal the molecular basis for soybean maturity locus E1 that regulates photoperiodic flowering. *Proc. Natl. Acad. Sci. U.S.A.* 109 E2155–E2164. 10.1073/pnas.1117982109 22619331 PMC3420212

[B65] Yang C. Xu Z. Song J. Conner K. Vizcay Barrena G. Wilson Z. (2007). Arabidopsis MYB26/MALE STERILE35 regulates secondary thickening in the endothecium and is essential for anther dehiscence. *Plant Cell* 19 534–548. 10.1105/tpc.106.046391 17329564 PMC1867336

[B66] Yang H. Xue Q. Zhang Z. Du J. Yu D. Huang F. (2018). *Arabidopsis* GmMYB181, a soybean R2R3-MYB protein, increases branch number in transgenic. *Front. Plant Sci.* 9:1027. 10.3389/fpls.2018.01027 30065741 PMC6056663

[B67] Zhang Y. Zhao L. Li H. Gao Y. Li Y. Wu X. (2013). GmGBP1, a homolog of human ski interacting protein in soybean, regulates flowering and stress tolerance in *Arabidopsis*. *BMC Plant Biol.* 13:21. 10.1186/1471-2229-13-21 23388059 PMC3571917

[B68] Zhang Z. Zhu J. Gao J. Wang C. Li H. Li H. (2007). Transcription factor AtMYB103 is required for anther development by regulating tapetum development, callose dissolution and exine formation in *Arabidopsis*. *Plant J.* 52 528–538. 10.1111/j.1365-313X.2007.03254.x 17727613

[B69] Zhao L. Li M. Xu C. Yang X. Li D. Zhao X. (2018). Natural variation in GmGBP1 promoter affects photoperiod control of flowering time and maturity in soybean. *Plant J.* 96 147–162. 10.1111/tpj.14025 30004144

[B70] Zhao L. Wang Z. Lu Q. Wang P. Li Y. Lv Q. (2013). Overexpression of a GmGBP1 ortholog of soybean enhances the responses to flowering, stem elongation and heat tolerance in transgenic tobaccos. *Plant Mol. Biol.* 82 279–299. 10.1007/s11103-013-0062-z 23636865

[B71] Zhong X. Dai X. Xv J. Wu H. Liu B. Li H. (2012). Cloning and expression analysis of GmGAL1, SOC1 homolog gene in soybean. *Mol. Biol. Rep.* 39 6967–6974. 22350155 10.1007/s11033-012-1524-0

