# Supplementary material for: Overexpression of GmGAMYB Accelerates the Transition to Flowering and Increases Plant Height in Soybean
Source: Front Plant Sci. 2021 May 10;12:667242. doi: 10.3389/fpls.2021.667242 (PMC8141843; doi:10.3389/fpls.2021.667242)
Supplement: Supplementary file 2 [file Data_Sheet_2.pdf]

Supplementary **Figure 1.** Staining of organs of *proGmGAMYB::GUS Arabidopsis*. The stem leaves, inflorescence, rosette leaves and roots of 30-day-old *proGmGAMYB::GUS Arabidopsis* plants were placed into X-Gluc staining solution. Took photos after decoloring.

Supplementary **Figure 2.** Molecular identification of *GmGAMYB-ox* transgenic soybean. (A) Identification of *GmGAMYB-ox* transgenic soybean by PCR analysis. (B) The expression levels of *GmGAMYB* were quantified by qRT-PCR analysis in transgenic soybean and WT plants. *GmActin4* gene was used for normalization. For each experiment, three technical replicates were conducted. Data shown are mean ± SD of three independent experiments (**P < 0.01, Student's t-test).

Table S1. Plant *GAMYB* genes' information.

Gene name	Accession number
GmGAMYB	KC525897
MpMYB33	RDX95167.1
SsGAMYB	TKY68413.1
CcMYB33	XP_020219565.1
VuMYB33	XP_027909434.1
VaGAMYB	XP_017431637.1
LaGAMYB	XP_019449331.1
AtMYB65	NP_001327042.1
AtMYB33	sp|Q8W1W6.1
OsGAMYB	LOC_Os01g59660.1
ZmGAMYB	NP_001130632.1
VvGAMYB	CBI40642.3
MeGAMYB	Manes.04G153700.1
SlGAMYB	Solyc01g009070.2.1
CsGAMYB	Cucsa.364400.1
HvGAMYB	HORVU3Hr1G079490.4
MtMYB33	XP_013445158.1
PtrMYB157	Potri.003G189700.1
RcGAMYB	29686.m000890

Table S2. The specific sequences of the primers.

Primer	Sequence (5'-3')
GmGAMYB-3F6H-F	TGGAGCTCGGTACCCATGAAGAAAGATATTGAAGATG
GmGAMYB-3F6H-R	GATCCTGGGATCCCCGAGAGGGGCTGGAATGGATTTTCA
GmGAMYB-TOPO-F	CACCATGAAGAAAGATATTGAAGATG
GmGAMYB-TOPO-R	GAGGGGCTGGAATGGATTTTCA
proGmGAMYB-GUS-F	CACCATAAGTAACGAGGCAGTGTAAT
proGmGAMYB-GUS-R	AACAAGAACGAACGCTAGAAAC
qGmGAMYB-F	GCGGCTGATTGCTGAACTT
qGmGAMYB -R	GTGCTTTGGCTATGCTGAC
qGmGA20ox-F	GATAGAGAGACCCTGTGCCT
qGmGA20ox-R	TGAGAAGCAGAGCAAAACAGAG
qGmFULc-F	GAAAACATTCCCTTCGGTTGAA
qGmFULc-R	TGATGATGAAGACTGATTCGCT
qGmFUL1a-F	AAAAATTCATAGACGGTGTCGC
qGmFUL1a-R	GGACAATTGGTAGTTTTTGGCT
qGmFUL2b-F	GCTTGTGATAGATTCAGCACAG
qGmFUL2b-R	CCTGCTTTGTTGCTAGTACATC
qGmTCP8-F	ATTTGCACGTAGTCCAATAACG
qGmTCP8-R	CAGCTTGTTCTTTCACACGTAA
qGmTCP12-F	CTTCGATCAATTCACATGCGAT
qGmTCP12-R	TTGTTGTTGGAAGTAGAGGGTT
qGmFPF1-F	CAAGGAAACCCATCTTTCTGTG
qGmFPF1-R	CAATCGTGAATTGCTAGAGCTC
qGmActin4 -F	GTGTCAGCCATACTGTCCCCATTT
qGmActin4-R	GTTTCAAGCTCTTGCTCGTAATCA
GmGBP1-TOPO-F	CACCATGGCCACTCTGAAAGAGCTTC
GmGBP1-TOPO-R	GAATGCCCTCTTTCAAATCCAATGC
