# Supplementary figures and images for: Overexpression of GmGAMYB Accelerates the Transition to Flowering and Increases Plant Height in Soybean
Source: Front Plant Sci. 2021 May 10;12:667242. doi: 10.3389/fpls.2021.667242 (PMC8141843; doi:10.3389/fpls.2021.667242)

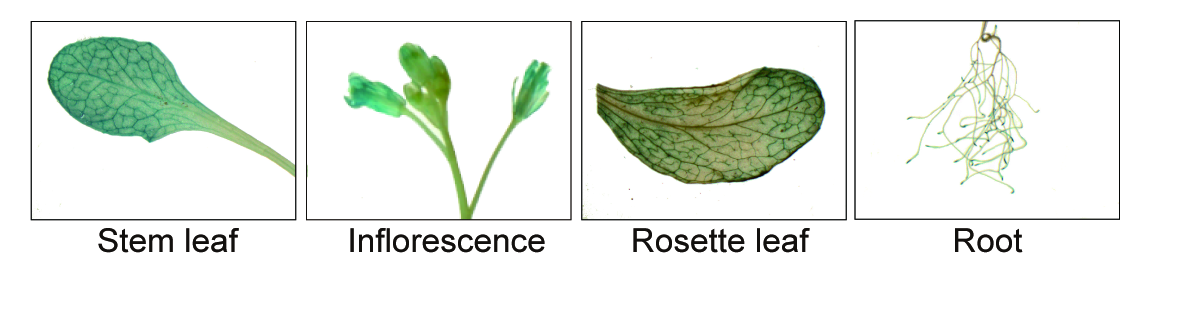

Supplement: Supplementary file 3 [file Image_1.TIF]

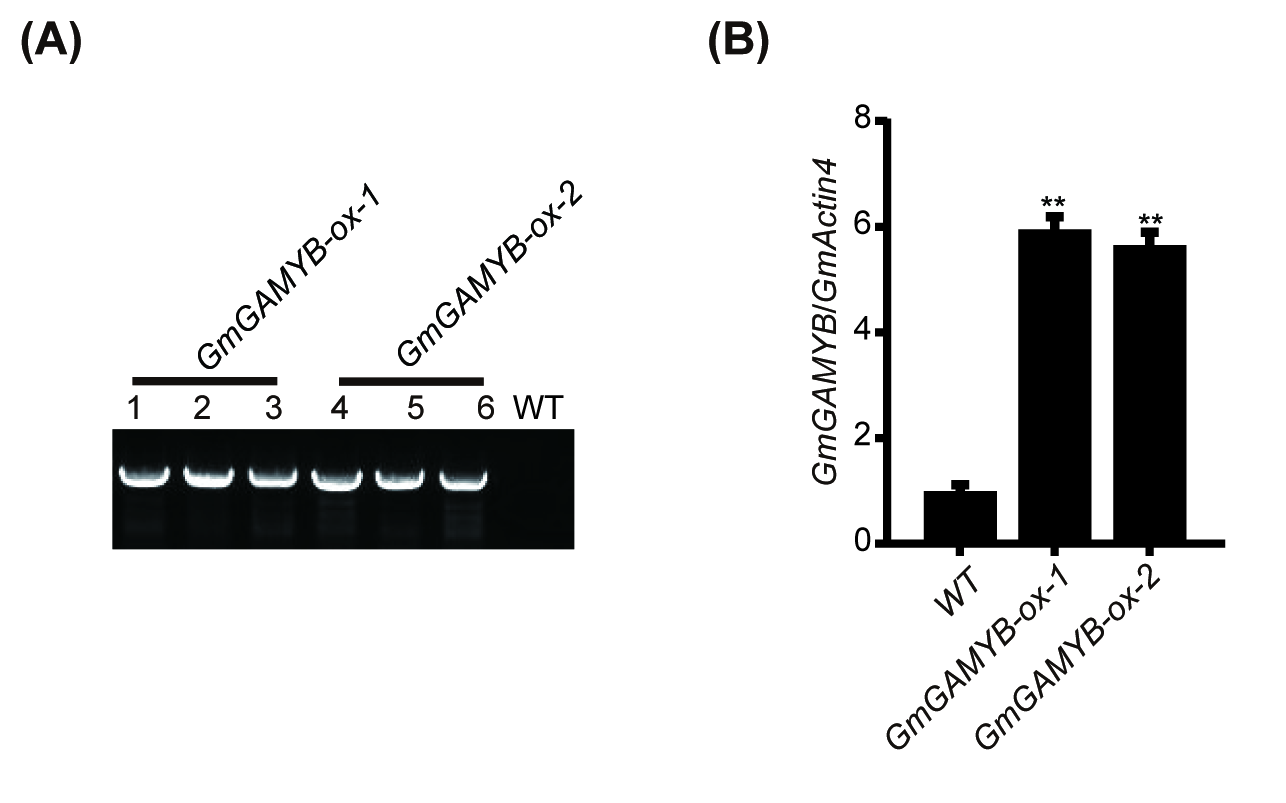

Supplement: Supplementary file 4 [file Image_2.TIF]
